# Acute Pyelonephritis and an Incidental Diagnosis

**DOI:** 10.7759/cureus.54446

**Published:** 2024-02-19

**Authors:** Inês Santos, Maria I Simão, Maria J Lúcio, Miguel O Santos, Guilherme Sacramento, Catarina I Cabral, Joana Vaz

**Affiliations:** 1 Internal Medicine, Hospital Egas Moniz, Lisbon, PRT; 2 Gastroenterology, Hospital Egas Moniz, Lisbon, PRT; 3 Pulmonology, Hospital Egas Moniz, Lisbon, PRT; 4 Oncology, Instituto Português de Oncologia de Lisboa Francisco Gentil, Lisbon, PRT; 5 Internal Medicine, Unidade Local de Saúde de Matosinhos, Matosinhos, PRT

**Keywords:** external bowel obstruction, acute obstructive pyelonephritis, gastrointestinal stromal tumor, omental stromal tumor, extra gastrointestinal stromal tumor

## Abstract

Gastrointestinal stromal tumors (GISTs) arise from the gastrointestinal tract. In rare cases, extra-gastrointestinal stromal tumors (EGISTs) occur in the omentum, mesentery, et cetera. They are mostly asymptomatic or have unspecific symptoms. Risk stratification classification systems are based on tumor size, mitotic rate, location, and perforation. The gold standard for diagnosis is a computed tomography (CT) scan. Ultrasound/CT-guided percutaneous biopsy allows histopathology and immunochemistry results (most stain positive for CD117 (c-KIT), CD34, and/or DOG1). Mutational analysis (most are in proto-oncogene c-KIT and platelet-derived growth factor receptor A (PDGFRA)) determines appropriate therapy. Surgical resection is the gold standard of treatment, with adjuvant and neoadjuvant molecular-targeted therapies depending on recurrence risk and mutations. This report describes a rare case of GIST (omentum EGIST) with a rare presentation (acute pyelonephritis) in a 67-year-old woman. Abdominal examination showed tenderness and a positive Murphy sign on the left side. Blood analysis presented microcytic hypochromic anemia, aggravated renal function, leukocytosis, and increased C-reactive protein. Abdominal CT revealed a heterogeneous abdominal mass, and a CT-guided biopsy showed epithelioid cells positive for CD117 and DOG1, which is compatible with a GIST. The patient underwent surgery that determined the GIST's origin from the greater omentum. Histology revealed an epithelioid GIST with large dimensions and a high histologic grade. Genetic testing detected a variant in the *PDGFRA* gene. With a high risk of progression, the patient received a three-year course of imatinib.

## Introduction

Extra-gastrointestinal stromal tumors (EGISTs) are rare and mostly asymptomatic or appear with unspecific symptoms depending on their location [1,2]. To the best of the authors' knowledge, acute obstructive pyelonephritis has not been reported as a presentation of EGISTs. Compared to GISTs, EGISTs usually have a worse prognosis [2]. The gold standard of treatment is surgical or endoscopic resection and mutational analyses determine appropriate molecular-targeted therapies [1,3,4].

## Case presentation

A 67-year-old Caucasian female presented to the emergency department with a history of fever, chills, left lumbar pain, and constipation. Four days prior to presentation, the patient was diagnosed with acute pyelonephritis and treated with ciprofloxacin but showed no improvement. Her past medical history included chronic obstructive pulmonary disease, hypertension, chronic kidney disease, type 2 diabetes mellitus, primary hypothyroidism, erosive gastritis, and she had breast cancer one year before the present description.

**Figure 1:**
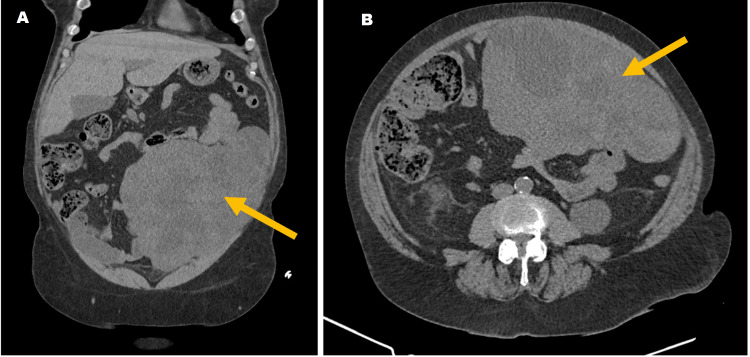
Abdominal heterogeneous mass on CT scan (yellow arrow) in a coronal plane (A) and transverse plane (B).

On physical examination, the patient was alert and fully oriented. Her vital signs were stable, and she was afebrile. Abdominal examination showed tenderness and a positive renal Murphy sign on the left side. Blood analysis presented microcytic hypochromic anemia (hemoglobin 8.5 g/dL), aggravated renal function (creatinine 2.12 mg/dl), leukocytosis (22,200 cells/uL), and increased C-reactive protein (20.8 mg/dL). A computed tomography (CT) of the abdomen and pelvis was performed and revealed a heterogeneous abdominal mass (215 x 127 x 126 mm) arising from the small bowel wall (Figure 1). It also showed signs of pyelonephritis in the left kidney and perinephric stranding. Acute obstructive pyelonephritis was the presumptive diagnosis.

Due to the possibility of malignancy, the patient was admitted for further investigation. Carcinoembryonic antigen and cancer antigen 19-9 levels were normal. Histology of the CT-guided biopsy of the mass revealed epithelioid cells positive for CD117 and DOG1, which is compatible with a GIST. As for the pyelonephritis, urine and blood cultures were negative. The patient completed a seven-day course of ceftriaxone with a good clinical and analytical response, and she was referred for surgical treatment. External bowel compression by the mass led to continuous constipation and the need for laxative treatment. Further investigation of the anemia revealed an iron and folate deficiency with no visible blood loss. Endoscopic studies were performed, finding two small duodenal angioectasia. However, the colonoscopy was incomplete due to pain in the transverse colon (associated with the mass). Red blood cell concentrates, ferric carboxymaltose, and folic acid were administered with a good response.

During surgery, surgeons determined that the origin of the mass was the greater omentum. A full resection of the mass was possible. However, there was no clear cleavage plane with the stomach, so a partial gastrectomy was performed. The histopathologic examination concluded that the mass was an epithelioid GIST of 36.5 cm (largest dimension) with pleomorphic cells, 50% necrosis, and a mitotic rate of 14/50 high-power field (HPF), which is considered a high histologic grade (G2). As the biopsy revealed, the immunohistochemical staining was positive for DOG1, CD117, and CD56. Genetic testing (polymerase chain reaction and bidirectional direct sequencing) was negative for KIT gene mutations but positive for a variant on the platelet-derived growth factor receptor A (*PDGFRA*) gene (p.Trp559_Arg560del) (Table 1). Fortunately, a gastric invasion was excluded (pT4N0). Nonetheless, these results determined a high risk of progression, so the patient was offered a three-year course of therapy with imatinib. So far, she has completed one year of treatment with no signs of recurrence.

**Table 1:** Immunohistochemistry and genetic panel.

	Immunohistochemical staining	Genetic testing (mutations)
Positive	DOG1, CD117, and CD56	PDGFRA gene (p.Trp559_Arg560del)
Negative	Chromogranin, S100, and AML	KIT gene

## Discussion

GISTs arise from the gastrointestinal (GI) tract (interstitial cells of Cajal) and are found in the stomach, small bowel, colon, rectum, and esophagus (in descending order of prevalence) [1]. Overall, GISTs account for 1-2% of GI neoplasms [1], being the most common mesenchymal neoplasms in the GI tract but are rare elsewhere [2]. In rare cases (< 5-10%), GISTs occur in the abdominal cavity, mostly in the omentum and mesentery, but also in the retroperitoneum, liver, mediastinum, pharynx, and gall bladder [2,3]. The median age at diagnosis is 65 years (a little inferior for EGISTs), with similar prevalence in men and women [1-3]. GISTs are mostly sporadic (97%) with no specific risk factors (besides some rare tumor syndromes) [3]. There are no specific symptoms, and they depend on the GIST's location (namely nausea, vomiting, abdominal distension, and pain) and size (for example, symptoms of obstruction such as dysphagia, jaundice, and constipation) [1]. In EGISTs, because they usually have more space available to grow, symptoms are even more delayed [2]. GISTs have a high rate of malignant transformation (10-30%), mostly among those not affecting the stomach, and exophytic growth is the most common [1]. Metastases occur in the liver, mesentery and omentum, lung, subcutaneous tissues, lymph nodes, and bone [1].

Many risk stratification classification systems have been created based on tumor size, mitotic rate, location, and perforation; they predict the need for neoadjuvant/adjuvant therapy [1]. The most common system is the tumor-grade metastasis system [1]. Comparing to GISTs, EGISTs usually have higher mitotic rates, a larger size, and distant metastasis, conferring a worse prognosis [2]. The gold standard for diagnosis is a CT scan; however, other imaging exams include ultrasound, magnetic resonance imaging, and positron emission transverse tomography [1,3,4]. Ultrasound/CT-guided percutaneous biopsy contributes to a definitive diagnosis with histopathology (histologic types are spindle (70%), epithelioid (20%), and mixed (10%)) and immunochemistry (most GISTs stain positive for CD117 (c-KIT), CD34, and/or DOG1) [1,4]. Mutational analyses determine appropriate therapy [3]. Most mutations occur in *KIT* (75%) and *PDGFRA* (10-20%) genes, while 5-10% have other mutations (*BRAF*, *NTRK*, etc.) [3,4]. Approximately 40-50% of EGISTs have *KIT* or *PDGFRA* mutations [2].

Besides surgical or endoscopic resection (the gold standard of treatment depending on tumor size and location), there are molecular-targeted therapies (for instance, selective tyrosine kinase receptor inhibitors such as imatinib) because traditional chemotherapy and radiation are ineffective [1,4]. Treatment strategies depend on recurrence risk and mutations. Resection of the high-risk primary tumor is followed by 400 mg of imatinib daily for three years [4], as decided for our patient. The optimal follow-up strategy is unknown [3,4]. Patients in high-risk categories usually undergo follow-up with CT scans every three to six months for three years during adjuvant therapy, then every three months for two years followed by every six months until five years after ending adjuvant therapy and every year for five additional years [4]. 

## Conclusions

This case has been reported due to an unusual presentation (acute pyelonephritis) of a rare type of GIST (omentum EGIST). The patient was considered to have a GIST with a high risk of progression and a variant of the *PDGFRA* gene. She underwent surgery followed by imatinib. After one year of therapy, there is no evidence of recurrence.
